# Reading Fluency in Children and Adolescents Who Stutter

**DOI:** 10.3390/brainsci11121595

**Published:** 2021-11-30

**Authors:** Mona Franke, Philip Hoole, Ramona Schreier, Simone Falk

**Affiliations:** 1 Institute of Phonetics and Speech Processing, LMU, 80799 Munich, Germany; hoole@phonetik.uni-muenchen.de (P.H.); Ramona.Schreier@phonetik.uni-muenchen.de (R.S.); 2 Department of Linguistics and Translation, UdeM, Montréal, QC H3T 1J4, Canada; simone.falk@umontreal.ca; 3 International Laboratory for Brain, Music and Sound Research (BRAMS), Montréal, QC H2V 2S9, Canada

**Keywords:** stuttering, reading fluency, reading accuracy, articulation rate, prosodic phrasing, fluent pauses, breath pauses

## Abstract

Speech fluency is a major challenge for young persons who stutter. Reading aloud, in particular, puts high demands on fluency, not only regarding online text decoding and articulation, but also in terms of prosodic performance. A written text has to be segmented into a number of prosodic phrases with appropriate breaks. The present study examines to what extent reading fluency (decoding ability, articulation rate, and prosodic phrasing) may be altered in children (9–12 years) and adolescents (13–17 years) who stutter compared to matched control participants. Read speech of 52 children and adolescents who do and do not stutter was analyzed. Children and adolescents who stutter did not differ from their matched control groups regarding reading accuracy and articulation rate. However, children who stutter produced shorter pauses than their matched peers. Results on prosodic phrasing showed that children who stutter produced more major phrases than the control group and more intermediate phrases than adolescents who stutter. Participants who stutter also displayed a higher number of breath pauses. Generally, the number of disfluencies during reading was related to slower articulation rates and more prosodic boundaries. Furthermore, we found age-related changes in general measures of reading fluency (decoding ability and articulation rate), as well as the overall strength of prosodic boundaries and number of breath pauses. This study provides evidence for developmental stages in prosodic phrasing as well as for alterations in reading fluency in children who stutter.

## 1. Introduction

Reading aloud is a competence that is built up in the early school years and accompanies us through adolescence, and even into adulthood, for example during scholarly activities, public speeches, or when we read to younger children. Reading aloud requires a number of cognitive capacities (e.g., working memory, attention), literacy skills (e.g., phonological awareness, orthographic awareness, morphological awareness, morphosyntactic knowledge, letter name knowledge, vocabulary, semantic processes, and grammatical knowledge), and comprehension [1]. It also requires advanced speech (motor) skills which, in the literature, are summarized under the term “reading fluency” [2]. Reading fluency includes online text decoding (i.e., a measure of reading accuracy), fluent articulation (i.e., reading rate representative of reading speed and automaticity), and meaningful prosodic performance (associated with meaningful text structuring) [2,3]. Hence, reading aloud can be a challenging activity for children and even adolescents who are still in the process of acquiring all these skills.

Reading aloud, and in particular, speech motor components pertaining to reading fluency, pose a particular challenge for individuals who suffer from disruptions of fluent speech production, such as stuttering. Stuttering is a neurodevelopmental speech motor disorder [4] and about 5–9% of children and adolescents are affected by developmental stuttering, which develops mostly between the ages of 2 and 3.5 years [5,6]. In one percent of the population, it persists into adolescence and adulthood, whereby males are four times more affected than females [5]. During childhood, the male-to-female ratio is more balanced, indicating that girls are more likely to recover from developmental stuttering than boys [5]. Remission of stuttering is most likely in the first years after stuttering onset, as a longitudinal study from Yairi and Ambrose [7] shows. Hence, persistence of stuttering becomes more and more likely with rising age, and especially during adolescence, remissions occur more rarely [5,7]. The speech of persons who stutter, whether read or spontaneous, can be described as frequently interrupted by repetitions or prolongations of sounds, syllables, or words, as well as hesitations and unvoluntary stops [8]. These stuttering symptoms greatly disturb the temporal flow of speech. Moreover, an increased temporal motor variability is characteristic of stuttering and was found in verbal and non-verbal tasks for children, adolescents, and adults who stutter [9,10,11]. Even the perceptually fluent speech of persons who stutter is temporally distinct from speech in persons with typically fluent speech. For example, adults who stutter show slower speech rates, and longer voice onset times, stop gap durations, vowel durations, or consonant-vowel transition durations in perceptually fluent speech (see [12], for a review). Articulatory measures reveal longer movement durations and longer durations from movement onset to peak velocity as well as longer temporal intervals between articulatory and phonatory events, resulting in longer voice onset times compared to typically fluent persons [13,14,15,16]. Furthermore, children who stutter (henceforth, CWS) in the age range from 4 years to 5 years 11 months show reduced amplitudes and velocities of articulatory movement [17] and a higher variability in oral motor coordination [18].

While previous studies have mostly focused on how reading fluency is acquired in typically fluent children (henceforth, CTF) in early school years, fewer studies have reported on CWS (see also Section 1.2). As reading fluency continues to develop in later school years, in particular rate and also prosodic aspects (see Section 1.1 below), there is a need for more studies on older children and adolescents [3,19]. Moreover, to our knowledge, no study has examined reading fluency in older CWS or adolescents who stutter (henceforth, AWS). One reason for this lack of research may lie in the fact that standardized tests for reading fluency do not necessarily mirror reading abilities of CWS or AWS (see [20], for a summary). Hence, it is hard to compare reading performances to typically fluent speaking peers and detect whether students who stutter have deficits in reading fluency.

As stuttering considerably alters the temporal flow of speech, it is likely to continue to disturb reading fluency throughout later school years, with the potential to diminish CWS’s and AWS’s school and social success (e.g., during presentations, participation in class involving reading aloud, artistic performances, etc.) [21]. Hence, the general aim of the present study is to shed light on alterations in rate and certain prosodic aspects of reading fluency experienced by pre-adolescent children and adolescents who stutter compared to typically fluent peers.

### 1.1. Development of Reading Fluency

With regard to the development of decoding ability, when children are between 7 and 9 years old, they are typically in the stage of a decoding reader [22], indicating that they are in a state of semi-fluency. They are yet unable to produce unknown words correctly. Children between the ages of 9 and 15 years are considered to be fluent and comprehending readers, until they reach the expert level of fluency, which typically happens by 16 years of age [22].

For reading accuracy, not much improvement has been found for children between 11 and 13 years (from 6th to 8th grade), as displayed in the American “National Oral Reading Fluency Norms” [23]. A similar result was found in a longitudinal study on German-speaking school-aged children, as there was no significant difference in accuracy between students at around 9–10 years of age and 13–14 years of age. However, in the same study, reading rate increased significantly between those age groups (from 174.5 to 267.2 syllables per minute) [24].

Reading rate increases during development and reaches its maximum in young adulthood (20–39 years) until it decreases again from the age of 40 [25]. In [25], children (8–12 years) had a reading rate of 144 words per minute (wpm), teenagers (13–19 years) 190 wpm, young adults (20–39 years) 193 wpm, and adults (older than 40) had a reading rate of 164 wpm [25]. However, this is only one study, so we do not know how general the finding is and as stated by Hasbrouck and Tindal [23], norms of reading rate usually do not go beyond grade 8. Hence, there is a lack of norms for the secondary and college levels.

As to reading prosody, Godde and colleagues [3] reviewed 13 studies that addressed reading prosody, mainly in English-speaking children in primary school. Meaningful reading prosody builds on several components, such as the correct placement and marking of stress, boundary accents, pauses and pre-pausal lengthening, phrase segmentation, and adjustment of phrase length [3]. One particular challenge in acquiring meaningful reading is the process of structuring a text into meaningful ‘chunks’. This process is called prosodic phrasing [26], by which a complex utterance is divided into segments [27]. Prosodic phrasing, which is the focus of our analysis on prosody, is phonetically signaled through phrase-final lengthening, pitch patterns, and the placement of pauses [28]. The position and duration of pauses play an important part in this segmentation process [3]. In fact, pause placement leads to great variability in reading style among readers [3]. Inter-and intra-sentential pauses decrease with age, as reading rate speeds up [3]. Pause durations decrease from 7–8-year-old students to 12–13-year-old students and increase again until adulthood [29].

In children of around 8–9 years of age who begin to read faster, placement of respiratory pauses becomes more random since the need to breathe is more important than taking grammar into account [3]. As Godde, Bosse, and Bailly [29] point out, the ability of using pausing patterns properly develops with age. By the age of approximately 13 years, children are able to produce pause patterns in an adult-like way. The frequency of breath pauses decreases from children at the age of around 8 years to children at the age of approximately 13 years. The latter age group had a similar frequency of breath pauses as adults. Interestingly, children around 8 years of age as well as adults produce significantly longer breath pauses compared to children between the ages of 9 and 13 years. Godde et al. [29] argue that younger students (approximately 8-year-olds) produce longer pauses due to difficulties in reading and that longer pauses give them more time to decode the text, while adults produce longer pauses for linguistic and expressive purposes. Adults mark major syntactic boundaries by making longer pauses and they also show more variability in pause durations, which leads to the assumption that adult readers read more expressively [29]. In sum, the present literature suggests that pre-adolescents between 8/9 and 12 years of age may differ, at least, in their pause patterns, and potentially, text segmentation from adolescents 13 years and older.

### 1.2. Stuttering and Aspects of Reading Fluency

As in other speech motor disorders, speech rate has been extensively studied with relation to stuttering. Lowering rate is a powerful tool in order to reduce stuttering symptoms in spontaneous as well as read speech ([30], for a summary). For instance, “prolonged speech” is one of the most familiar and most effective speaking techniques used in fluency-shaping therapy ([31], for a summary). When using this technique, persons stretch vowels, consonants, and/or syllables and they use a continuous airflow and/or light articulatory contacts [30,31]. Speaking techniques that only focus on speech rate are also referred to as stretched syllables, controlled rate, or slow or smooth speech [31]. Hence, a lower rate may be used by individuals who stutter to control and reduce overt stuttering symptoms.

In CWS, results regarding reading rate point towards slower rates, although results on non-read speech do not always find differences with children who do not stutter. In a one-minute reading task, school-aged CWS produced fewer words compared to CTF [32]. However, [32] did not provide information on how stuttering symptoms during the task were handled. Slower oral reading rates (fluent syllables per minute) were also found in adults who stutter [33] and school-age (8–11 years old) CWS [34]. Bosshardt [35] reported slower articulation rates in fluent read speech in both children from 6 years of age to 10 years of age and adults who stutter. Other results on general articulation rate (i.e., fluent conversational or narrative speech), measured in syllables per second, showed no differences between school-aged CWS and CTF between the ages of 5 years 6 months and 10 years 7 months [36], nor between 7 years 3 months and 12 years 7 months [37].

Concerning prosody, only a few studies addressed the question whether prosody in read speech shows alterations in individuals who stutter. Bergmann [38] reported a higher variability in the timing structure of stressed intervals in male adults who stutter compared to typically fluent adults, measured as the distance between pitch frequency peaks on vowels in fluent passages of a read poem. This result led the author to the assumption that stuttering might comprise “a prosodic disturbance, specifically (as) a motoric difficulty in producing stressed syllables” [38] (p. 297). A study on pauses in oral reading in adults who stutter showed that they produced significantly more pauses in the range between 150 and 250 ms [39]. These results suggest that at least adults who stutter may experience some difficulties in generating prosodic structure.

Only two studies so far have examined prosody production in CWS, and only one of them in read speech. Meyers Fosnot and Jun [40] investigated prosodic characteristics (i.e., pitch range and duration, type of pitch accents, boundary tones) in CWS and children with autism in the age range between 7 and 14 years in short sentences using reading and imitation tasks. The speech of CWS showed similar patterns compared with the control group. CWS did not differ significantly from children in the control group, in terms of phrase durations and pitch range [40]. However, with four children per group, the study had a very small sample size and further investigation with a bigger sample size is needed to elaborate potential differences between young persons who stutter and typically fluent peers in the prosody of reading. The other study from Arbisi-Kelm, Hollister, Zebrowski, and Gupta [41] focused on spontaneous speech production with 12 CWS and 12 CTF. The authors found that CWS produced a narrower pitch range across utterance types, but they did not differ from the control group in terms of utterance duration. However, CWS produced a greater degree of pre-boundary lengthening preceding relative clauses in syntactically complex sentences, as well as higher fundamental frequency variability at these juncture points [41].

In sum, the current evidence is too sparse to decide whether rate or prosodic aspects of reading fluency show alterations in CWS or AWS.

### 1.3. Aims of the Present Study

Young readers who stutter may display decreased reading fluency compared to their peers, not only because of overt stuttering symptoms, but also because of altered performance in rate and pause management and, potentially as a consequence, in text segmentation (prosodic phrasing). As the above literature shows, there is a lack of studies investigating these aspects of reading fluency in CWS and AWS, and also their fluent peers. According to previous studies, a change in reading fluency, particular in pause management and potentially text segmentation, is expected between pre-adolescence (9–12) and adolescence (13–17, adult-like performance) in typically fluent readers, but it is unclear if stuttering could delay or even hinder some of these changes from occurring. Hence, the aim of the present study was to compare children (9–12) and adolescents (13–17) who do and do not stutter with regard to these aspects of reading fluency.

We recorded 52 participants, 26 German-speaking children and adolescents who stutter and 26 typically fluent peers in the age range of 9 to 17 years while reading a chapter of a children’s book aloud. Using the same text for all participants has the advantage that the content and (syntactic) complexity of the text was constant across participants. While we did not expect to find differences in reading accuracy (decoding ability) between the groups, we expected to find an age effect because children are still in the process of becoming expert readers (they reach expert levels by approximately the age of 16 [22]). Furthermore, children are still in the process of becoming fluent speakers with a mature speech motor system [42]. For this reason, we hypothesize a slower articulation rate, longer pauses, and more prosodic phrases in children compared to adolescents.

Slower articulation rates and longer and more pauses during reading are also expected in CWS and AWS compared to their matched peers [35]. As a consequence of rate and pause management, CWS and AWS could chunk the text into smaller units and thereby produce a higher number of prosodic phrases. Previous results with adults point in this direction because phrases of adults who stutter included less words compared to typically fluent persons in a storytelling task [43].

## 2. Materials and Methods

### 2.1. Participants

Twenty-six German children (CWS) and adolescents who stutter (AWS), diagnosed by speech therapists, and a control group consisting of 26 age- and gender-matched German children (CTF) and adolescents with typically fluent speech (ATF) participated in the study. The age ranges for the children and adolescent groups are as follows:

Children between 9 and 12 years constituted the younger group (n = 11 males, 2 females, mean age (CWS) = 10.65 years (SD = 1.06), mean age (CTF) = 10.88 years (SD = 1.23)) and adolescents from 13 to 17 years constituted the older group (n = 11 males, 2 females, mean age (AWS) = 14.97 years (SD = 1.13), mean age (ATF) = 14.82 years (SD = 0.95)). The participants had no cognitive, language, or attentional impairment. Five AWS, two ATF, and two CWS were multi- or bilingual. The participants who stutter were recruited through the intensive therapy course “Stärker als Stottern” (staerker-als-stottern.de), during which their stuttering was assessed by trained speech therapists. The typically fluent participants were recruited through schools. The stuttering severity of each participant who stutters was determined with the SSI-3 by trained speech therapists on the recording day (see Table 1). All participants who stutter had received therapy before they participated in this study. Six participants who stutter reported to have read with a fluency-enhancing technique (e.g., slowed speech rate, gentle speech initiation, soft voice onset) or a stutter modification technique which is conscious stuttering without concomitant behaviors (e.g., conscious repetitions of word onsets). These fluency-shaping techniques were either used locally or globally. Participants’ reports were confirmed by a speech therapist. All participants gave their informed consent for inclusion before they participated in this study. The study was conducted in accordance with the Declaration of Helsinki, and the protocol was approved by the Ethics Committee of the medical faculty of the Ludwig Maximilian University of Munich.

### 2.2. Stimuli

The participants were asked to read an excerpt from a popular German children’s book recommended for readers from 8 years on [44] which was printed on two DIN A4 pages (Times New Roman, 12.5). The excerpt contained narrative parts (narrator, 3rd person perspective) as well as passages of direct speech which rendered the reading more engaging. The excerpt contained 690 words (1058 syllables).

### 2.3. Procedure

Participants were comfortably seated at a table and were asked to read the text in a lively manner, at their own preferred tempo. Note that readers read the text aloud once they received their copy without preparing for it (i.e., no silent reading before starting to read aloud).

Participants were recorded with a ZOOM H4N recorder (44.1 kHz, 16 bit), via an external headset microphone (beyerdynamic opus 54.16/3), in a quiet room with the examiner present in the room. The duration of one recording session varied depending on the reading performance of the participant and if they had read the complete excerpt. Recording durations ranged from 2.36 to 12.96 min. Note, that there were 10 participants who stutter (5 CWS and 5 AWS) who were not able to read the complete text due to severe stuttering or time constraints.

### 2.4. Analyses

In order to measure decoding ability and temporal aspects of reading, a one-minute period of the text was chosen (i.e., situated in the first quarter of the full excerpt). On average, all participants produced 199 syllables in one minute (SD = 57.43, min = 57, max = 300). We did not use an initial passage as this could have increased reading errors and disfluencies that would have been caused by the initial excitement the young participants may have experienced in a recording session. A passage in the first quarter was also more appropriate in case a participant was unable to continue reading until the end of the excerpt (e.g., in case of severe stuttering). For more detailed phrasing analyses, a short excerpt of this one-minute period was chosen that consisted of 3 sentences (39 syllables). The excerpt contained direct speech and narrative, which is representative for the text type (i.e., children’s book) and was analyzed for all participants.

#### 2.4.1. Decoding Ability

Decoding ability relates to the accuracy of reading. It is defined (in percent) as the number of read words without errors in 60 s, divided by the overall number of words read per 60 s multiplied by 100 [45]. Errors included omissions, additions, mispronunciations, replacements, and reversals that had not been corrected by the reader. The overall number of read words was defined as the number of printed words a participant read in 60 s. If there was an omission of a word in the text excerpt, it counted as an error within the read words. However, repeated words, as well as self-corrections, did not count as an error and did not count towards the read words per minute [45]. Therefore, stuttering events did not count as errors in this method. The decoding ability in percent gives information about the proficiency of reading. According to Rasinski [45], an advanced reading level (“independent reader”) is reached if 96 to 100% of the words are correctly pronounced, and an accuracy level from 90 to 95% is considered as being adequate [2].

#### 2.4.2. Articulation Rate and Pauses

An orthographic transcription of the one-minute period was made. Together with the corresponding sound file, the files were processed via the “Pipeline without Automatic Speech Recognition”, a tool from the Bavarian Archive for Speech Signals (BAS) Services [46,47]. Inter-word pauses were automatically segmented, with a minimum duration of 50 ms. The output (i.e., TextGrids compatible with the program Praat [48]) that were generated from the Pipeline were manually checked and corrected if the syllables did not match the speech signal.

Stutter events and other disfluencies were annotated in the TextGrids for the one-minute period by trained speech therapists following the rules of the SSI diagnosis [49] based on audio recordings without video. Repetitions of syllables (but not words) were considered as stuttering events, whereby repetitions counted as one stutter event. In general, monosyllabic word repetitions were not analyzed as a stutter symptom, unless the words were uttered with a markedly increased tonus or speech rate. In the latter case, every repetition counted as a stutter event. Filler words, such as “uhm” or “ah” that were clearly intended to inhibit or delay a stutter event counted as stutter events, too [49]. Finally, syllables after silence (blockade) were marked as a stuttering event. Trained speech therapists detected and marked all pauses that were caused by disfluencies (“disfluent pauses”).

Non-stuttering-specific disfluencies, referred to here as “other disfluencies”, such as interjections, mispronunciations, as well as repetitions of words or phrases were also detected. Together, stutter events and other disfluencies are called disfluencies. The number of disfluencies per participants was calculated with the following formula:

Disfluent syllables (in %) = ((number of disfluent syllables)/(number of all syllables)) × 100(1)

These segmentations/annotations were used to determine articulation rate as a measure of reading rate. We used articulation rate and not speech rate because the latter measure usually includes disfluencies and pauses which are shorter than 1–2 s (see [50], for a summary). Therefore, speech rate would only display stuttering symptoms. On the other hand, articulation rate indicates how fast syllables are produced, based on fluent speech. Both velocity changes and amplitude changes of speech movements affect articulation rate. It is therefore considered an important measure to evaluate articulatory coordination and development [51,52]. Pauses are considered separately. Articulation rate was defined by the following formula:

Articulation rate = number of fluent syllables/total duration of excerpt − duration of disfluencies (syllables + pauses) − pauses > 250 ms(2)

Note that pauses above 250 ms were excluded in the articulation rate calculation similarly to previous studies (for example [50,52,53]).

Finally, mean pause durations of all “fluent pauses” were extracted per participant.

#### 2.4.3. Prosodic Phrasing

The prosodic analysis was performed using conventions for identifying prosodic phrases from the German version of the ToBI framework (Tones and Break Indices), a system for the annotation of spoken prosody [54]. Note that ToBI is a model following the autosegmental-metrical approach in phonology [55,56,57], and therefore provides a sophisticated tool to study cues to phonological intonational structure. However, the present analysis was only focused on segmentation (the “breaks” part), using phonetic markers that we define in Table 2 (partially adapted from GToBI, [54]), and without the intention to investigate the bases of phonological phrase structure. This analysis includes more detailed information about phrasal boundaries than, e.g., pauses alone. Although pauses and pause durations reveal general information about the temporal structuring of a text, phrases can also be produced without a pause and in particular, children may use diverse markers to segmentation. These markers are displayed in Table 2. Furthermore, as can be seen in Table 2, on the basis of these markers, GToBI advances a categorical classification of breaks into major and minor boundaries, potentially reflecting perceptual processes.

In Table 2, there are four label options: one for a major (label “4”) intonation phrase (IP) boundaries, and three for minor boundaries, i.e., intermediate (label “3”) phrase (ip) boundaries (both are also present in the mainstream American English ToBI system, e.g., [57]), and two smaller breaks defined by tonal and rhythmic structure (these labels are specific to the German ToBI system [54]). The label “2r” is used for a rhythmic break with tonal continuity and the label “2t” is used for a tonal break with rhythmic continuity [54].

One IP includes at least one ip but can also include more (e.g., [54]). The phrasal type depends on the degrees of juncture or boundary strength. While the intermediate phrase has lower boundary strength (e.g., phrase accent with no or only small pauses), the intonational phrase has higher strength (e.g., phrase accent and boundary tone with a longer pause) [57].

**Figure 1:**
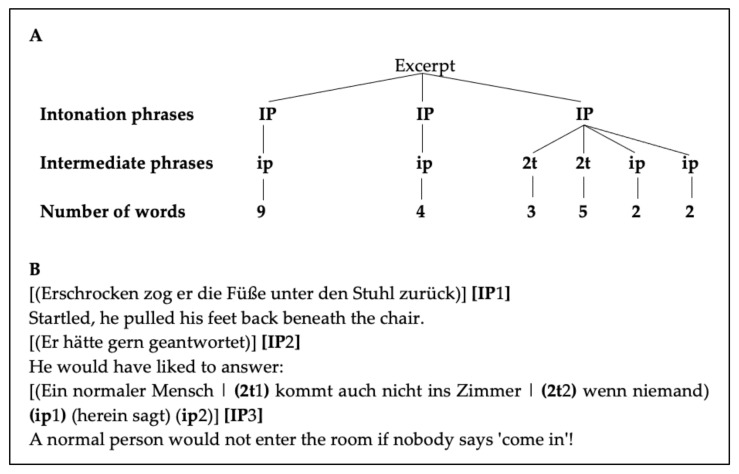
(**A**): Phrasing of the excerpt by a professional audiobook reader [58]. (**B**): Corresponding phrasing excerpt with English translation of the excerpt.

For the phrasing analysis, a text excerpt of the 1-min reading period was chosen. The phrasal structure was identified in each participant’s rendition of the short excerpt based on conventions displayed in Table 2, using Praat [48]. As an example, Figure 1 displays the prosodic phrasing analysis of the excerpt from the performance of a professional audiobook reader [58]. We included the professional reader’s performance on prosodic phrasing and articulation rate, as well as pause duration, in the analysis in order to have a model of the best possible meaningful text segmentation.

In all the analyses, “disfluent pauses” were excluded from the analysis. For example, when a blockade resulted in a particularly long pause preceding a word, the interval before the word was identified as a pause that was caused by a disfluency. Thus, this pause did not count as a marker of a phrase boundary.

We then calculated the overall break index to have a general measure of prosodic phrasing. It takes the number of phrases and the boundary strength into account by adding all individual break indices. Thus, the overall break index is constituted by the sum of all IP (=4), ip (=3), 2r (=2), and 2t (=2) break indices that had been produced within the phrasing excerpt. Finally, the number of IPs and ips were also taken into account in order to get a more detailed picture of the composition of the break index.

## 3. Results

The first part of this section reports results based on analyses of the one-minute reading period, starting with descriptive information about the number of disfluencies, followed by results on reading accuracy (decoding ability), articulation rate, and fluent pause durations.

The second part of the analysis presents results of the more qualitative prosodic phrasing analyses according to the autosegmental-metrical model approach (GToBI) on the shorter excerpt within the 1-min-period, including results on the overall break index (and its correlation with disfluencies), as well as major and minor prosodic phrases.

All statistical analyses were conducted in RStudio (version 4.0.2). For group comparisons, a series of two-way ANOVAs (type III sums of squares) were performed for the dependent variables decoding ability and break index with the between-subject factors stuttering (group who stutters vs. group who does not stutter) and age (children vs. adolescents). To gain more insights into individual stuttering behavior and reading, Spearman-rho correlations were calculated between these variables and the number of actual disfluencies in the read passage (in percent). Finally, to evaluate reading maturity, comparisons with a professional adult reader were done for each group (age group +/− stuttering).

Normal distribution of the data was checked with the Kolmogorov–Smirnov test, and the homogeneity of groups was checked with Levene’s test before running each analysis. Non-parametric tests were used for evaluating articulation rate, the GToBI phrasing analysis and whenever the assumption of normality was not met. Finally, a linear regression was performed in order to examine different parameters of reading fluency and their potential impact on prosodic phrasing in young speakers who do and do not stutter.

### 3.1. Disfluencices

Table 3 displays participants’ disfluencies in the analyzed reading excerpts. In both the larger and smaller excerpt, the group who stutters clearly shows more disfluencies compared to the group who does not stutter, as expected. AWS had more disfluencies than CWS and were more variable. ATF were most fluent.

### 3.2. Decoding Ability

**Figure 2:**
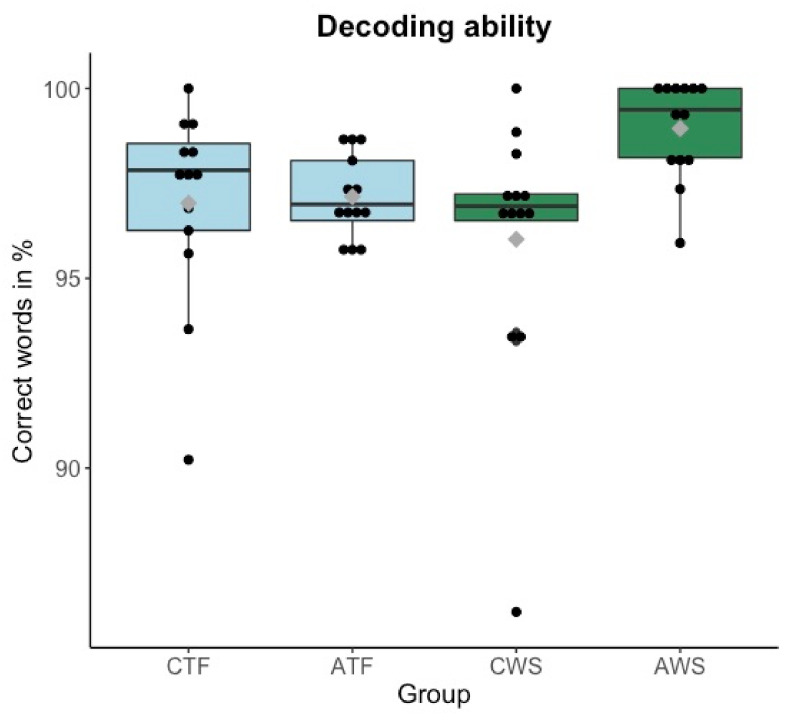
Boxplots of decoding ability-percentage of correctly produced words per group in a one-minute reading period (y-axis). Each dot represents each participant’s reading accuracy in percent. Mean values per group are visualized with a gray diamond within each boxplot. CTF: children with typically fluent speech, ATF: adolescents with typically fluent speech, CWS: children who stutter, AWS: adolescents who stutter.

Results on decoding ability indicate that reading per se was not a problem for the participants. Most of the participants, as displayed in Figure 2, are “independent readers” (between 96 and 100% accuracy) and a few are “adequate readers” (between 90 and 95% accuracy), according to Rasinski’s [2,45] classification of reading level. This means that almost all participants read more than 90% of the text correctly. Nevertheless, there is one value below 90%, which signals that the frustration level in this case should be very high [44]. This participant was removed for the further analysis of decoding ability.

An ANOVA was run (as described earlier) and revealed that there was no effect of stuttering (F(1,47) = 2.63, *p* = 0.112). Decoding ability slightly increased in the older compared to the younger age group, which is also reflected by the mean values of 96.92% vs. 98.05% accuracy (F(1,47) = 4.90, *p* = 0.032). No interaction was found (*p* = 0.067).

### 3.3. Articulation Rate

**Figure 3:**
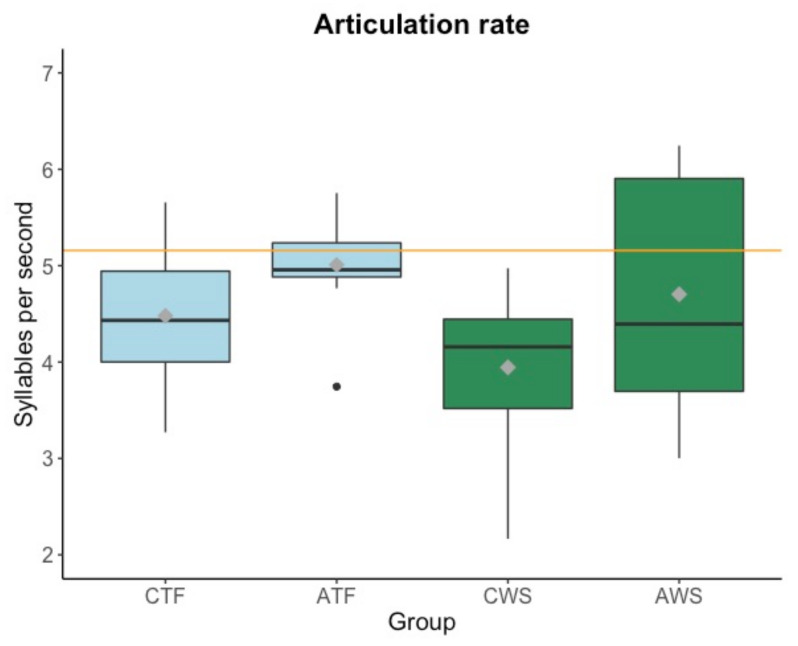
Boxplots of articulation rate in syllables per second (without disfluencies, disfluent pauses, and pauses > 250 ms) (y-axis). Mean values per group are visualized with a gray diamond within each boxplot. Articulation rate of a professional audiobook reader is displayed as a reference with an orange horizontal line. CTF: children with typically fluent speech, ATF: adolescents with typically fluent speech, CWS: children who stutter, AWS: adolescents who stutter.

Articulation rate (see Figure 3) varied substantially among participants ranging from 2.17 to 6.25 syllables per second.

As can be seen in Figure 3, the group of AWS in particular showed a wide range of articulation rates, indicating that a long history of stuttering may lead to more variable outcomes in articulation rate during reading. In absence of group homogeneity of variance, Welch two-sample *t*-tests were run for each age group, which revealed no significant effect of stuttering in children (*t* = 1.901, df = 23.96, *p* = 0.069), nor in adolescents (*t* = 0.870, df = 16.023, *p* = 0.397) on articulation rate.

We then compared the mean articulation rate of each group (CTF, ATF, CWS, AWS) to the articulation rate of the professional audiobook reader. Bonferroni-corrected results suggest an effect of age, that is, children read significantly slower than the audiobook reader (CWS: *p* < 0.001; CTF: *p* = 0.01) while adolescents did not differ from the professional reader.

**Figure 4:**
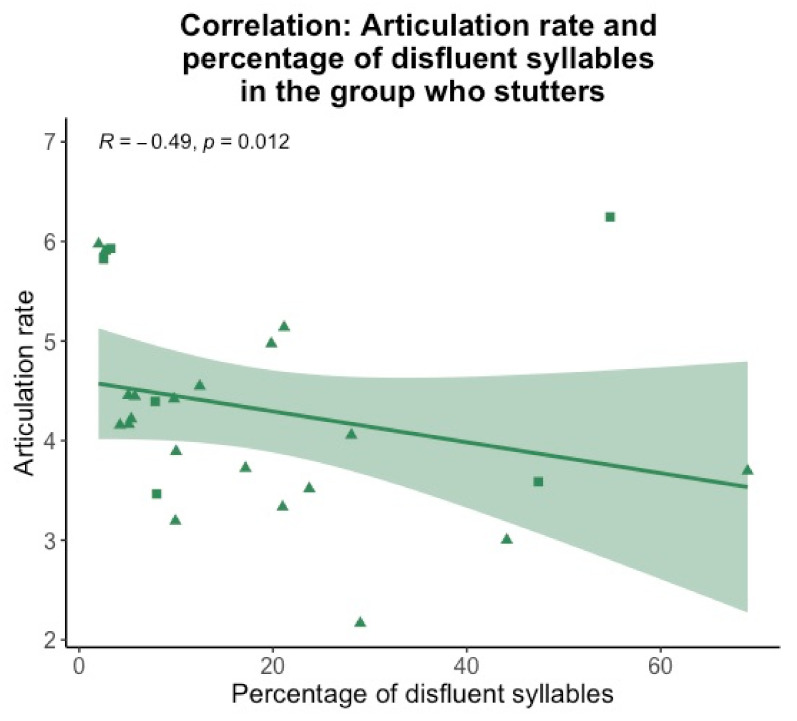
Spearman-rho correlation for the articulation rate (y-axis) and percentage of disfluent syllables (x-axis) for the group who stutters. Triangles display participants who did not read with a fluency-shaping technique, squares display participants who did read with a fluency-shaping technique.

To examine whether disfluencies, although excluded in the calculation, affected articulation rate, we calculated a Spearman-rho correlation between these two variables for participants who stutter and participants who do not stutter. The percentage of disfluencies negatively correlated with the articulation rate in the group who stutters (R = −0.49, *p* = 0.012, Figure 4), but not in the control group (R = −0.24, *p* = 0.24). That is, the more disfluencies participants who stutter produced, the slower their articulation rate. The exclusion of the participants reading with a fluency-shaping technique did not change this result. Hence, participants who stutter may have chosen their articulation rate as a function of produced (or anticipated) disfluencies.

### 3.4. Pause Durations

**Figure 5:**
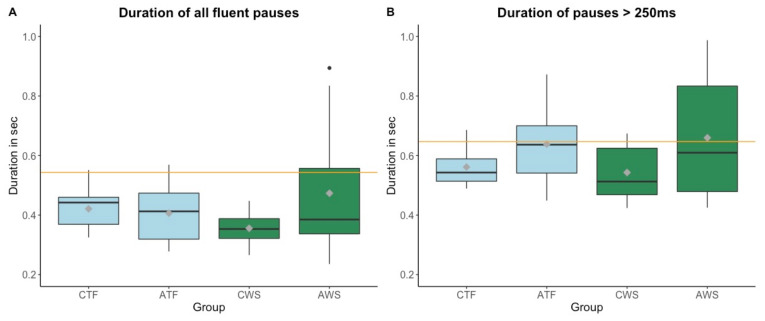
Boxplots of duration of all fluent pauses (**A**) and pauses above 250 ms (**B**) per group in a one-minute reading period (y-axis). Mean values per group are visualized with a gray diamond within each boxplot. Pause duration of a professional audiobook reader is displayed as a reference with an orange horizontal line. CTF: children with typically fluent speech, ATF: adolescents with typically fluent speech, CWS: children who stutter, AWS: adolescents who stutter.

Figure 5 shows the pause durations per group for all pauses (including longer pauses) and longer pauses (>250 ms) separately in the one-minute reading excerpt.

In absence of group homogeneity of variance, Welch two-sample *t*-tests were used to examine whether there was an effect of stuttering in the younger and the older group on pause durations. Children and adolescents were analyzed separately in order to avoid a potential confound with age and related articulation rate changes (see above). Results on all fluent pauses revealed a significant effect of stuttering in children (*t* = 2.557, df = 23.238, *p* = 0.018) but not in adolescents (*t* = −1.433, df = 15.056, *p* = 0.172). That is, CWS displayed overall shorter pause durations than CTF. No significant effect of stuttering was found for longer pauses (>250 ms).

We then compared pause durations of each group (CTF, ATF, CWS, AWS) to pause durations of the professional audiobook reader. Bonferroni-corrected results revealed that children in particular displayed shorter pauses than the audiobook reader (all fluent pauses: CWS: *p* < 0.001, CTF: *p* < 0.001; longer pauses: CWS: *p* = 0.004; CTF: *p* = 0.001). ATF produced slightly shorter pauses only when taking all fluent pauses into account (*p* = 0.001).

To examine whether disfluent speech and stuttering affected pause durations, similar to articulation rate, we correlated pause durations with the percentage of disfluencies. However, no significant effects were found.

### 3.5. Prosodic Phrasing

For two AWS, it was impossible to decide whether the phrasing was only conditioned by severe stuttering. Therefore, we excluded these two participants from the phrasing analyses, as well as the participant with low decoding ability. Table 4 displays the mean number of produced phrases (IP, ip, 2r, 2t) per group, after these exclusions.

#### 3.5.1. Overall Break Index

**Figure 6:**
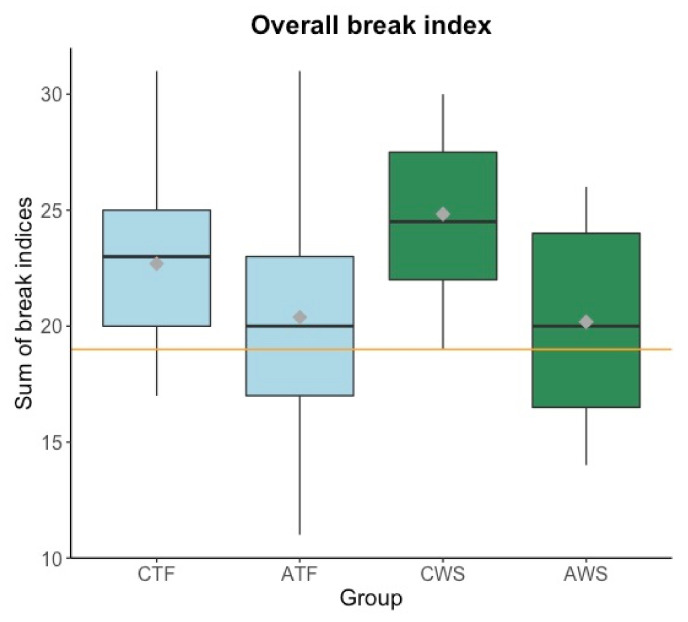
Boxplots displaying the overall break index of the phrasing in the excerpt (y-axis). Mean values per group are visualized with a gray diamond within each boxplot. The overall break index of a professional audiobook reader is displayed as a reference with an orange horizontal line. CTF: children with typically fluent speech, ATF: adolescents with typically fluent speech, CWS: children who stutter, AWS: adolescents who stutter.

First, we calculated the overall break index (see Figure 6), a measure that includes overall boundary strength and number of phrases. As the overall break index conflates minor and major boundaries, a higher break index can either originate from a higher number of major boundaries or a higher number of phrases in general. To disentangle these two options, more detailed analyses, especially on major boundaries, are reported below.

An ANOVA was performed (as described at the beginning of the result section) and results revealed that there was no effect of stuttering (F(1,45) = 0.62, *p* = 0.44). However, the overall break index decreased in the older compared to the younger age group (main effect of age: F(1,45) = 7.97, *p* = 0.007), that is, older participants produced overall fewer or fewer major phrase boundaries than younger participants.

A one-sample *t*-test was run for each group (CTF, ATF, CWS, AWS) in order to compare its mean overall break index to the overall break index of the professional audiobook reader. Bonferroni corrected results revealed that the overall break index of CTF (*t* = 3.508, df = 12, *p* = 0.017) and CWS (*t* = 5.337, df = 11, *p* < 0.001) was significantly higher compared to the audiobook reader’s overall break index. The two adolescent groups did not differ from the professional reader.

To examine whether disfluencies affected the overall break index, we calculated a correlation between these two variables. The percentage of disfluencies positively correlated with the overall break index in the group who stutters (R = 0.48, *p* = 0.019), but not in the control group (R = 0.067, *p* = 0.74). That is, the more disfluencies participants who stutter produced, the higher their overall break index. This result applied to all participants who stutter but not when excluding the participants who used a fluency-shaping technique (R = 0.34, *p* = 0.17).

**Figure 7:**
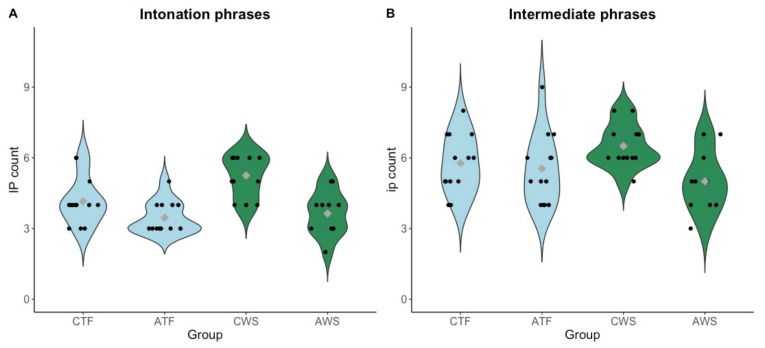
Violin plots for each group, gray diamonds displaying the mean values per group. (**A**): Sum of produced intonation phrases (IPs) per excerpt, each dot represents one participant. (**B**): Sum of produced intermediate phrases (ips) per excerpt, each dot represents one participant. CTF: children with typically fluent speech, ATF: adolescents with typically fluent speech, CWS: children who stutter, AWS: adolescents who stutter.

Second, in order to investigate the composition of the break index in more detail, major (IP) and minor phrase (ip) boundaries were examined separately (see Figure 7).

#### 3.5.2. Intonation Phrases

In absence of a normal distribution of the data, a Kruskal–Wallis test was used to examine the differences in produced IPs according to the group combinations (CTF, ATF, CWS, AWS). A significant difference (χ^2^ = 18.975, df = 3, *p* < 0.001) was found among the four groups. To determine whether there was an effect of stuttering on the number of produced IPs in each age group (children vs. adolescents), four Mann–Whitney U tests, first within each age group and then within each test and control group were run (Bonferroni-corrected α = 0.0125).

Results revealed that CWS produced significantly more IPs compared to CTF (W = 33, *p* = 0.043). For adolescents, no significant effect of stuttering on the number of produced IPs was found.

Age (children vs. adolescents) reduced the number of IPs in both groups; in the group who stutters significantly (W = 15, *p* = 0.005) from a mean of 5.25 (SD = 0.87) in children to 3.64 (SD = 0.92) in adolescents, and in the control group from a mean of 4.15 (SD = 0.99) in children to 3.46 (SD = 0.66) in adolescents.

One of the acoustic markers that was used to define an IP was a breath pause situated at the end of a phrase. Thus, the number of produced IPs highly correlated with the amount of breath pauses (R = 0.73, *p* < 0.001), although an IP can also be realized without a following breath pause. To gain more insight into the acoustic realization of IPs, we conducted further analyses on breath pauses (number and durations) in the phrasing excerpt.

An ANOVA (see details at the beginning of the result section) revealed that there was a significant effect of stuttering (F(1,45) = 8.31, *p* = 0.006) and a significant age group effect (F(1,45) = 35.37, *p* < 0.001) on the number of produced breath pauses, but no interaction. These results indicate that the group who stutters produced more breath pauses during the phrasing excerpt compared to the control group. A similar result holds for children compared to adolescents.

In addition, we checked whether the groups (CTF, ATF, CWS, AWS) differed in the mean duration of all breath pauses, as taking longer breath pauses might have been more important for the participants who stutter than for the typically fluent group. A Kruskal–Wallis test did not reveal differences between the groups.

#### 3.5.3. Intermediate Phrases

Intermediate phrases were analyzed in the same way as intonation phrases. The four groups (CTF, ATF, CWS, AWS) differed from each other (Chi-squared = 8.396, df = 3, *p* = 0.040). Subsequent Mann–Whitney U tests did not reveal a significant difference between CWS and CTF nor AWS and ATF. However, children and adolescents differed in the group who stutters (W = 23, *p* = 0.029) with CWS producing more ips than AWS.

#### 3.5.4. Prosodic Phrasing and Predicting Variables

Further, we wanted to establish how different parameters of reading fluency may impact prosodic phrasing in young speakers who do and do not stutter. We used the overall break index to display prosodic phrasing, as it takes the number and strength of all prosodic boundaries into account. For this purpose, we fitted a linear regression model to the data with overall break index (mean-centered) as the dependent variable and the mean-centered continuous variables articulation rate, amount of breath pauses, fluent-pause duration, number of disfluencies in the phrasing excerpt (with the interaction of group), and age in months as predictors.

The best fit model (F(4,44) = 12.04, *p* < 0.001, adjusted R-squared: 0.479, BIC = 274.247) contained articulation rate (estimated β = −3.080, *p* < 0.001) and number of breath pauses (estimated β = 1.015, *p* = 0.035) as the only significant predictors of the overall break index, as well as the duration of fluent pauses (estimated β = 3.954, *p* = 0.367), and number of disfluencies (estimated β = −1.129, *p* = 0.1) as non-significant predictors. Hence, across groups, prosodic phrasing was best predicted by articulation rate and number of breath pauses.

## 4. Discussion

The aim of this study was to investigate whether CWS and AWS differ from typically fluent peers in terms of reading fluency, including decoding ability, articulation rate, pausing, and prosodic phrasing. Another aim was to investigate differences in two different age groups—children (9–12 years) and adolescents (13–17 years). Analyses were based on participants’ naturalistic reading performances from a popular German children’s book to compare performances of exactly the same textual output.

Independently of stuttering, all participants except one were highly accurate readers. More than 90% of the one-minute excerpt was decoded correctly. Fluent articulation rate (excluding all disfluencies and pauses >250 ms from the analyses) did not differ between participants who do and do not stutter. However, as reported before for spontaneous speech [59], the number of disfluencies produced by a participant who stutters showed an impact on the (fluent) articulation rate, as participants with more disfluencies spoke more slowly. Furthermore, participants who stuttered displayed a higher number of breath pauses than their typically fluent peers. Although this result should be considered with caution, as it was obtained with a very short passage, breath management could be one of the crucial aspects to be investigated in future studies with regard to text segmentation.

CWS, but not AWS, showed a number of particularities in reading fluency and phrasing. First, they produced overall shorter fluent pauses and more major prosodic breaks (intonation phrases) than their typically fluent peers. They also produced more minor (intermediate phrases) prosodic breaks than AWS; an age effect that was not found in the typically fluent group. As to the overall prosodic segmentation (i.e., the overall break index), no significant group differences were found between participants who do and do not stutter. For all children and adolescents, independently of stuttering, articulation rate and the number of breath pauses were the best predictors for the overall break index, in line with previous literature [60]. This means that participants with slower articulation rates and a higher number of breath pauses chunked the text into more prosodic phrases (whether minor or major, as a strong correlation between the number of phrases and the overall break index shows, R = 0.91, *p* < 0.001) than participants who read faster, which resulted in a higher overall break index.

The results also show some age-related developmental patterns between 9 to 17 years of age. Overall, as suggested by [3,29], adolescents from 13 years on approached adult-like reading in several aspects. Decoding ability, although at a high level in all the participants, slightly improved between children (9–12 years) and adolescents (13–17 years). Articulation rate and pause durations reached adult-like rates in adolescents, as did the overall break index.

Children differed in all analyses from the adult model reader. They showed slower articulation rates and shorter pause durations. Compared to adolescents, they displayed shorter pause durations and more breath pauses.

Taken together, these findings support the assumption that an immature (children) or challenged (stuttering) speech motor system negatively affects the temporal aspects of reading fluency. Therefore, those who have to cope with the weakest speech motor system (i.e., CWS) are the most vulnerable to showing altered reading performance. Young persons who stutter may have to speak slower, take more breath pauses, and chunk the text into smaller units (i.e., resulting in more major prosodic boundaries, as in our study) in order to maintain reading fluency. This is a new result, as previously only adults who stutter were reported to build more/shorter phrases (i.e., phrases contained fewer words compared to phrases of typically fluent adults) when narrating a story [43].

Shorter pauses in children than in adolescents or the adult reader mirror previous findings on age-related changes (see [3,29]). However, CWS produced even shorter pauses than CTF, although the two groups did not greatly differ in the number of disfluencies (only ~8% more disfluencies in CWS compared to CTF). The difference in pause duration did not appear in longer (>250 ms) or breath pauses. Hence, it is a possibility that short pauses were driving the effect. In fact, one previous study found that persons who stutter produced significantly more pauses that are shorter than 250 ms in their fluent speech [39]. Potentially, CWS may have marked intermediate prosodic phrases with shorter pauses than their typically fluent peers or showed overall more similarities in pausing to even younger readers. Future studies could further investigate the exact nature and source of these aspects.

The above results suggest that CWS are confronted with particular challenges in reading. Some CWS may develop avoidance of reading aloud in class or in public and thus, may develop delays in reading fluency and more pronounced avoidance behavior. AWS seem to overcome some of those challenges at least when confronted with relatively easy texts (as in our study) to read. Although AWS had the highest number of disfluencies, they showed no significant differences compared to typically fluent speaking peers with respect to prosodic phrasing and overall fluent pause duration. CWS could also be more challenged by reading comprehension and text retelling, as reading prosody relates to these capacities [61,62], a potential avenue for future research. Hence, especially CWS should be supported and encouraged by educators and parents to read out loud.

The present results also suggest that, generally, higher rates of stuttering symptoms, independently of age, may cause some changes to reading, such as slowing down reading rate, altering breathing, and some aspects of prosodic segmentation.

It is another avenue for future research to investigate whether our results on phrasing in CWS also apply to conversational speech. Here, higher demands on speech planning, as for example in sentences with greater utterance length and/or higher sentence complexity could reveal differences among age groups and their prosodic capacities. Previous research found that, in adults who stutter, syntactic complexity negatively affected speech motor stability while utterance length did not [63]. However, the opposite was found for CWS (between the age of 7 and 12 years). Articulatory coordination in children worsened when the sentence length got longer, while syntactic complexity did not affect the speech motor coordination in CWS [37]. Nevertheless, syntactic complexity could particularly impact prosodic phrasing in CWS, as syntax and prosodic segmentation are deeply intertwined. Based on the present results on prosodic phrasing in read speech, one might predict that CWS would produce more prosodic phrases, particularly in more complex sentences, compared to AWS.

Higher cognitive effort could also induce higher sub-structuring and a higher number of phrases, whether in read or spontaneous speech. For example, it has been reported that adults who stutter produce shorter sentences in order to reduce the concurrent processes involved in speech planning and speech production (see [64] for a summary). General cognitive stress associated with reading or speaking might therefore affect all levels of processing, not only the articulatory execution.

From a neural perspective, future studies could focus on the development of subcortical speech motor control in young readers who stutter. For instance, the cerebellum plays an important role in articulatory motor control [65,66] and in developing reading skills [67,68] via a phonological circuit (dorsal-fronto-parietal pathway) and a semantic circuit (ventral fronto-temporal pathway) connected with the cerebellum [67]. Therefore, the cerebellum is hypothesized to influence phonological and word-based decoding as well as reading skill automatization (for a summary, see [67]). Adults who stutter, for example, display an increased activity in the cerebellum during the production of (silent and) overt speech which is associated with increased sensorimotor monitoring and lower automaticity in terms of speech motor skills [69]. Hence, we may expect an increased activity in the cerebellum of young readers who stutter while they read (syntactically/phonologically) more complex texts (or longer sentences), since this would increase the demands on speech motor performance (see [37]).

There were some limitations to the present study. One limitation is that the text excerpt for the prosodic analysis was relatively short and confined to one text type (a children’s book). This limitation was partly due to the fact that the prosodic analysis was very time-consuming because of the high variability of young readers in the use of acoustic cues and unpredictable sub-structuring (e.g., not respecting punctuation). Beyond children and adolescents, it would be interesting to analyze the variability of reading fluency in adults who do and adults who do not stutter. Since we only had one male professional reader as a reference for adult performance, an adult group who does not stutter would display greater variability and a more “standard” adult reading performance than a trained audiobook reader. Furthermore, future studies could compare prosodic phrasing in different text types and do more extensive text analyses. Future studies on conversational speech should include speech prosody, as well. It is another limitation that further research is needed to determine whether prosodic differences are due to ability or due to coping strategies.

In summary, the present study provides preliminary evidence for differences in reading fluency between young readers who stutter and their matched peers. Although participants who stutter did not have problems in decoding during reading and did not show significant differences in articulation rate, particularly CWS showed some alterations in prosodic phrasing and pause management compared to typically fluent peers; even in perceptually fluent speech. Hence, prosody should be taken into account when examining the reading performance of persons who stutter and may include a variety of other factors, such as reading comprehension, emotional stress, or articulatory measures.

## Figures and Tables

**Table 1:** Individual participant information at recording time. Children who stutter (CWS, upper part) and adolescents who stutter (AWS, lower part).

Participants Who Stutter	Sex	Age	SSI-3 Severity
P16	m	9	mild
P11	m	9	very severe
P19	m	9	severe
P18	m	9	very mild
P15	m	10	moderate
P04	m	11	mild
P13	m	11	very mild
P12	m	11	very mild
P10	m	11	very mild
P06	m	12	moderate
P26	f	12	mild
P14	m	12	moderate
P23	f	12	very mild
P17	m	13	severe
P70	m	14	very severe
P66	f	14	mild
P60	m	14	severe
P64	m	14	very severe
P51	m	15	very severe
P53	f	15	very mild
P62	m	15	mild
P56	m	16	mild
P61	m	16	very mild
P55	m	16	severe
P54	m	16	mild
P65	m	17	severe

**Table 2:** Criteria for phrasing analysis and tonal break assignment.

Break Index	Definition	Marker
4	Intonation phrase boundary	High boundary strength due to one or more of the following markers: - Breath pause - Silent pause accompanied by one or more of the following markers: - Creaky voice - Phrase-final lengthening - Pitch reset
3	Intermediate phrase boundary	Lower boundary strength due to: - Tonal and rhythmic break - Pause without breathing
2r	Rhythmic break with tonal continuity	Lowest boundary strength due to: - Hesitation pauses - Rhetorical pause - Unnatural lengthening of segments that is not attributable to the phrasal contour (e.g., phrase-final lengthening) [54]
2t	Tonal break with rhythmic continuity	Lowest boundary strength due to: Change in pitch level without a rhythmic disruption that is not attributable to the tonal contour of the phrase (often occurs in fast speech) [54]

**Table 3:** Mean percentage of disfluent syllables (SD in parentheses) per group in the excerpts relevant for the analyses.

Group	1-Min Reading Excerpt Mean (SD)	Excerpt of Prosodic Phrasing Analysis Mean (SD)
CTF	5.15% (4.82)	2.60% (3.16)
ATF	2.75% (2.81)	2.00% (5.23)
CWS	13.70% (8.93)	11.30% (8.34)
AWS	22.39% (20.11)	20.26% (20.11)

**Table 4:** Mean number of phrases and SD per group.

Group	Mean	SD
CTF	10.69	1.84
ATF	9.46	2.02
CWS	12.17	1.80
AWS	9.36	1.91

## Data Availability

The data presented in this study are available on request from the corresponding author. The data are not publicly available due to privacy reasons.
